# Non-Catalytic Functions of Pyk2 and Fyn Regulate Late Stage Adhesion in Human T Cells

**DOI:** 10.1371/journal.pone.0053011

**Published:** 2012-12-27

**Authors:** Nicole M. Chapman, Ashley N. Yoder, Jon C. D. Houtman

**Affiliations:** 1 Interdisciplinary Graduate Program in Immunology, University of Iowa, Iowa City, Iowa, United States of America; 2 Department of Microbiology, Carver College of Medicine, University of Iowa, Iowa City, Iowa, United States of America; University of Oslo, Norway

## Abstract

T cell activation drives the protective immune response against pathogens, but is also critical for the development of pathological diseases in humans. Cytoskeletal changes are required for downstream functions in T cells, including proliferation, cytokine production, migration, spreading, and adhesion. Therefore, investigating the molecular mechanism of cytoskeletal changes is crucial for understanding the induction of T cell-driven immune responses and for developing therapies to treat immune disorders related to aberrant T cell activation. In this study, we used a plate-bound adhesion assay that incorporated near-infrared imaging technology to address how TCR signaling drives human T cell adhesion. Interestingly, we observed that T cells have weak adhesion early after TCR activation and that binding to the plate was significantly enhanced 30–60 minutes after receptor activation. This late stage of adhesion was mediated by actin polymerization but was surprisingly not dependent upon Src family kinase activity. By contrast, the non-catalytic functions of the kinases Fyn and Pyk2 were required for late stage human T cell adhesion. These data reveal a novel TCR-induced signaling pathway that controls cellular adhesion independent of the canonical TCR signaling cascade driven by tyrosine kinase activity.

## Introduction

The engagement of the T cell antigen receptor (TCR) by a peptide-bound major histocompatibility complex is a crucial step in T cell activation and is accompanied by dynamic changes in the actin and microtubule cytoskeletons. Reorganization of the cytoskeleton is critical for T cell migration to secondary lymphoid organs and to sites of infection and inflammation [1], [2]. These cytoskeletal rearrangements also serve to enhance T cell adhesion to antigen presenting cells (APC) or infected target cells, a process that augments T cell effector functions [1], [2]. Blocking microtubule or actin dynamics prevents stabilized contacts with APC and inhibits T cell effector responses, including cytokine production, effector granule secretion, and proliferation [1]–[3]. Since T cell-APC interactions are compromised, humans with mutations in the actin nucleating protein WASp have defects in T cell activation, leading to enhanced susceptibility to infection and autoimmunity [4], [5]. Thus, appropriate T cell responses are intricately linked to cytoskeleton reorganization. Understanding how TCR signals control cytoskeletal dynamics may allow one to therapeutically target dysfunctional T cell activation linked to several human diseases [6], [7].

The earliest TCR signaling events that control actin cytoskeletal rearrangements have been extensively studied. TCR-dependent signaling is initiated by the Src family kinases (SFK), Lck and/or Fyn, which phosphorylate immunoreceptor tyrosine-based activation motifs (ITAMs) present in several TCR subunits. Following activation at these ITAMs, the tyrosine kinase ZAP-70 phosphorylates the adaptor proteins LAT and SLP-76, inducing downstream signaling events. These include the activation of the PI3 kinase (PI3K)/Akt and MEK kinase cascades and the induction of actin remodeling and nucleating proteins like Vav1, the Rho family GTPases, WAVE2, WASp, and Arp2/3 [2], [5], [8]. TCR-mediated actin polymerization is defective in the absence of SFK activity, LAT, or SLP-76 [1], [9]–[11], demonstrating that LAT complex formation is required for actin reorganization downstream of the TCR. TCR signals that drive adhesion within 20 minutes after receptor activation have also been shown to require the induction of these pathways [1], [2]. However, studies that examine how signaling at later times impacts adhesion are needed to better understand how actin remodeling promotes T cell activation.

Focal adhesion kinase (FAK) and proline-rich tyrosine kinase 2 (Pyk2) are two proteins that control the actin reorganization in numerous cell types and are phosphorylated downstream of adhesion receptors and the TCR [12]–[15]. The TCR-inducible phosphorylation of FAK is reduced in *fyn−/−* peripheral T cells [16], and the enzymatic activity of Lck and/or Fyn is needed for Pyk2 phosphorylation downstream of the TCR [17]–[19]. Therefore, Lck and Fyn may also initiate actin remodeling via FAK and Pyk2. Consistent with this idea, we recently found that Pyk2 phosphorylation induced by soluble anti-TCR treatment correlates with two separate bursts of actin polymerization in human T cells [18]. Pyk2 has also been demonstrated to co-localize with the microtubule organizing center (MTOC) in mouse CD8 T cells and is recruited to the APC-T cell contact site [20], [21]. Therefore, FAK and Pyk2 may be required for integrating TCR signals that drive cytoskeleton reorganization to promote human T cell adhesion.

The physiological ligand for the TCR is expressed on the surface of an APC. However, it is difficult to examine which TCR signaling pathways drive actin polymerization using APC-T cell conjugates, since co-stimulatory and adhesion receptors also induce intracellular signaling pathways that control actin rearrangement and cellular adhesion in these cells [1], [22], [23]. Therefore, the relative roles that TCR signaling proteins play in adhesion are still not entirely clear. Immobilized TCR-specific antibodies are a useful alternative for investigating which TCR signals control cytoskeletal dynamics. Like those cells stimulated with peptide-pulsed APC, T cells activated using plate-bound antibodies undergo morphological changes driven by the actin and microtubule cytoskeletons. These changes also require SFK, LAT, and SLP-76 activation, and TCR signaling is terminated upon treatment with actin de-polymerizing agents [1], [2], [9], [10]. Thus, the specific TCR signaling pathways that regulate adhesion can be investigated using plate-bound TCR-specific antibodies.

Here, we describe a near-infrared imaging technique to examine the TCR-mediated signaling events that control actin polymerization and adhesion in live human T cells. We found that TCR-mediated adhesion occurred in two unique phases in human T cells. While both of these adhesive events were driven by actin polymerization, the activity of Lck and/or Fyn was only required for the first wave of TCR-induced adhesion. We also show that Fyn and Pyk2 play non-enzymatic roles in controlling the second stage of TCR-dependent adhesion. These results demonstrate that TCR stimulation promotes two unique phases of adhesion in human T cells. Collectively, these data challenge the current paradigm that all TCR signals that drive adhesion emanate from proximal tyrosine kinase-dependent pathways.

## Results

### TCR activation promotes two phases of human T cell adhesion

**Figure 1:**
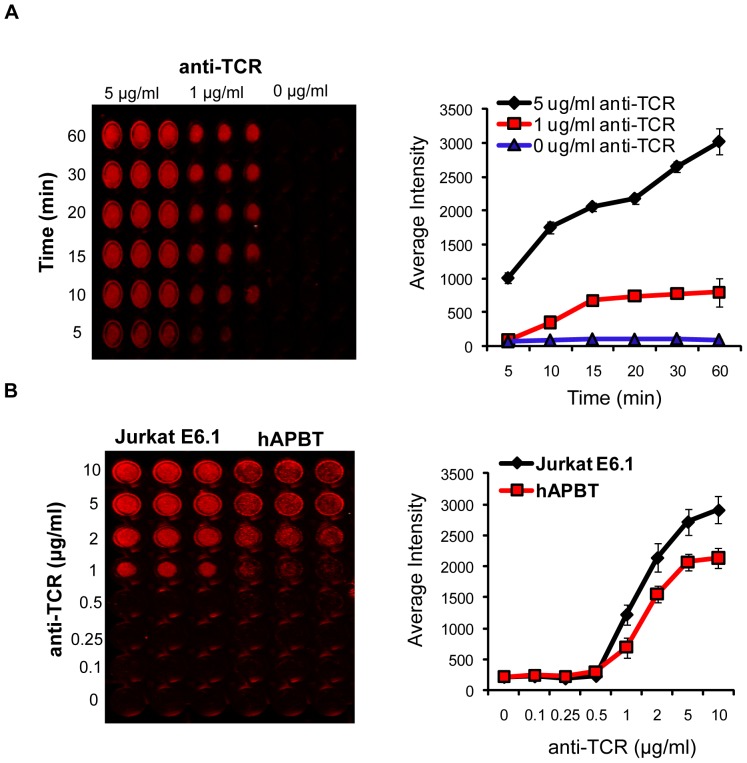
TCR activation promotes two phases of T cell adhesion. (A) Jurkat E6.1 were stained and incubated on an anti-TCR coated RIA/EIA plate for various times. The intensity of the membrane dye was measured to determine the relative number of cells that bound to each well. A representative plate is shown (left panel). The data were quantified and the average intensity of triplicate wells ± SD is shown graphically (right panel). The data are representative of three independent experiments. (B) Jurkat E6.1 cells and hAPBTs were stained and stimulated with various doses of immobilized anti-TCR for 30 minutes (left panel). Adhesion was measured and the average intensity of triplicate wells ± SD was graphed (right panel).

TCR induction leads to increased cellular adhesion [1], [2], yet the signaling events that drive TCR-mediated adhesion are still not completely understood. To address this question, we utilized an assay in which human T cells were activated on plates coated with stimulatory TCR-specific antibodies. Immobilized anti-TCR treatment was shown to induce Jurkat spreading and adhesion to antibody-coated surfaces [24]. To further investigate this phenomenon, the membranes of Jurkat cells were labeled with a fluorescent dye and were stimulated with immobilized anti-TCR for various times. The amount of cellular binding was then measured on the Licor Odyssey Infrared Imager. As shown in Figure 1A, no adhesion was detected in the absence of antibody (0 µg/ml: blue line), but weak, transient binding was observed within 5–10 minutes following activation with 1 µg/ml or 5 µg/ml anti-TCR. This adhesion stabilized within 20 minutes after TCR activation. Interestingly, Jurkat cells that were stimulated with 5 µg/ml of anti-TCR displayed even stronger adhesion at 30–60 minutes after activation, a finding that has not been reported previously using anti-TCR treated surfaces. Similar results were also observed when human activated peripheral blood T cells (hAPBTs) were stimulated with this concentration of anti-TCR for various times (data not shown). To further examine adhesion at this later time, we stimulated Jurkat cells or hAPBTs with various doses of immobilized anti-TCR for 30 minutes. As shown in Figure 1B, both Jurkat cells and hAPBTs bound to the plates following stimulation with 1–2 µg/ml of anti-TCR, with the maximal response occurring after activation with 5–10 µg/ml of the stimulatory antibody. These data show that continuous TCR activation correlates with enhanced adhesion and suggest that TCR stimulation may induce two distinct phases of adhesion in human T cells.

### Intracellular signaling and non-covalent interactions mediate TCR-induced adhesion to plastic plates

**Figure 2:**
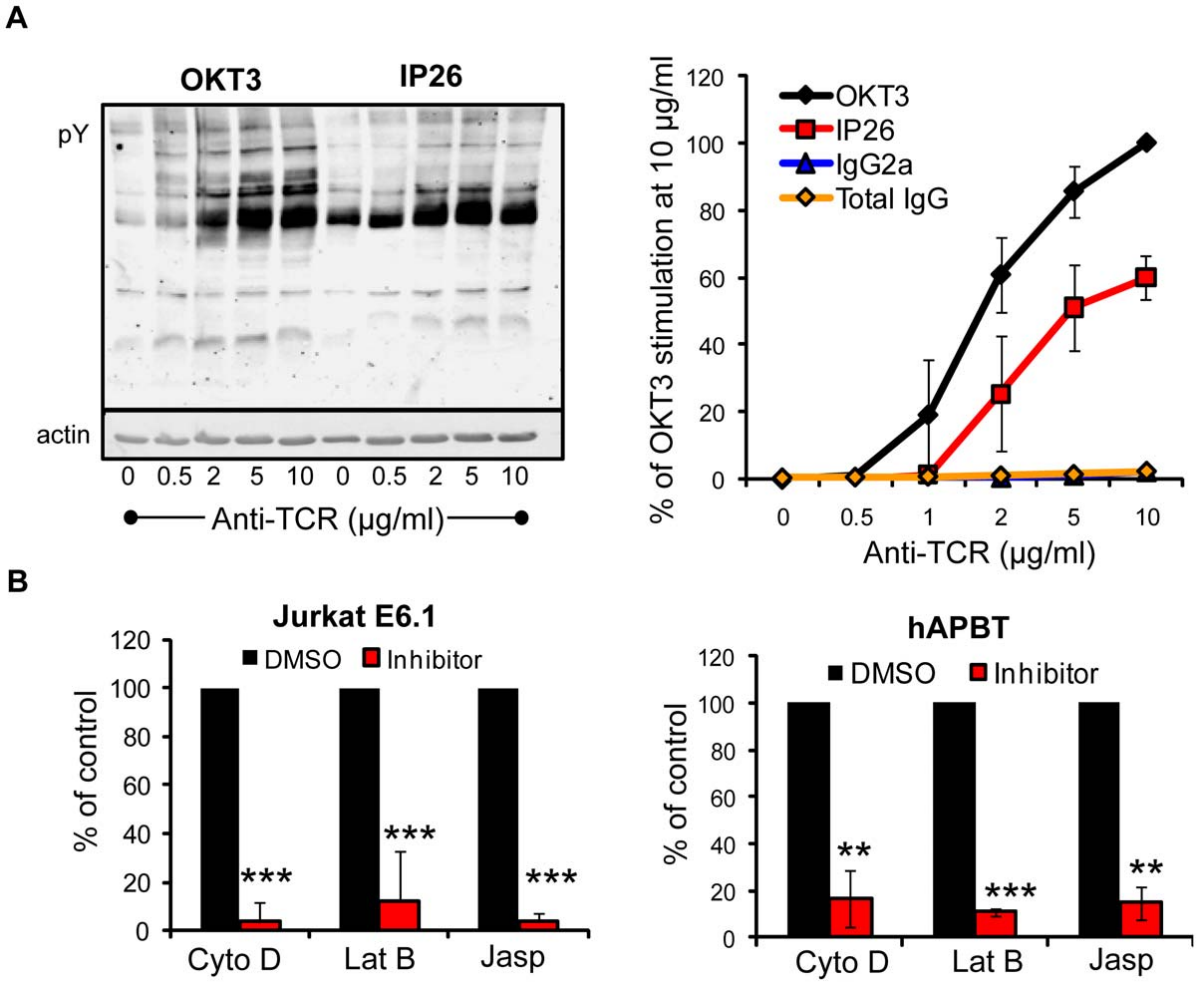
Intracellular signaling mediates adhesion to anti-TCR treated plates. (A) Jurkat E6.1 cells were stimulated with various concentrations of the immobilized anti-TCR antibodies OKT3 and IP26 for 30 minutes. Total changes in TCR-inducible tyrosine phosphorylation were detected by immunoblot (left panel). TCR-induced adhesion in response to these antibodies or two non-specific antibodies (IgG2a and total mouse IgG) was also quantified and is graphed as the average three experiments ± SD (right panel). These data are shown as the average of three experiments (B) Jurkat E6.1 cells (left panel) and hAPBTs (right panel) were stained and pretreated with DMSO or the actin cytoskeleton inhibitors Cytochalasin D (Cyto D) and Latrunculin B (Lat B) for 30 minutes or jasplakinolide (Jasp) for 15 minutes at 37°C. The cells were applied to an anti-TCR coated RIA/EIA plate and allowed to adhere for 30 minutes at 37°C. The data were normalized such that the DMSO samples were set to 100%. The normalized results from three independent experiments were then graphed. ** p≤0.01 *** p≤0.001.

Human T cells that were stimulated for greater than 30 minutes strongly adhered to anti-TCR-coated surfaces. To rule out the possibility that cells were binding to the F_c_ portions of the anti-TCR antibody via F_c_γ receptors, we treated the plates with the isotype control for the anti-TCR antibody OKT3 (IgG2a) or a non-specific total mouse IgG antibody (total IgG). We observed no binding to the plates when these non-specific antibodies were used (Figure 2A: right panel), demonstrating that adhesion was not driven by F_c_γ receptor binding to the F_c_ portions of the anti-TCR antibody.

It was also possible that this binding reaction was mediated by interactions between the plate-bound antibody and the TCR and not by antibody-induced intracellular signaling. To rule out this possibility, we compared the binding efficiency of Jurkat cells stimulated with two different agonistic anti-TCR antibodies. As illustrated by global changes in tyrosine phosphorylation, the anti-CD3ε antibody, OKT3, more strongly induced downstream signaling in Jurkat cells than an antibody directed against the αβ chains of the TCR, IP26 (Figure 2A: left panel). We found that weaker adhesion was observed when Jurkat cells were activated with the less stimulatory anti-TCR antibody (Figure 2A: right panel). Interestingly, the dose of antibody needed to achieve comparable levels of signaling and adhesion was 2–5 fold higher when IP26 was used to stimulate the cells (Figure 2A). Thus, adhesion to these anti-TCR-treated plates is dictated by the strength of intracellular signaling induced by the TCR.

We next wanted to confirm that intracellular signaling was regulating adhesion in this assay, since the different anti-TCR antibodies used above may have different affinities for the TCR that could lead to less adhesion. Both TCR-induced signaling and adhesion are linked to the actin cytoskeleton [1], [2]. Therefore, we predicted that if intracellular signaling was controlling adhesion, disruption of the actin polymerization should inhibit binding to these plates. By contrast, we would expect to see no effect on binding if the antibody-TCR association was controlling adhesion. As shown in Figure 2B, disrupting actin filaments with cytochalasin D and latrunculin B significantly abrogated the TCR-induced binding of Jurkat cells and hAPBTs to these plates. Cytochalasin D and latrunculin B could weaken the integrity of the T cell membrane, rendering these cells more susceptible to removal by washing. However, adhesion was also inhibited when cells were treated with jasplakinolide, a chemical that promotes actin polymerization and stabilizes actin filaments but is sufficient to block TCR-inducible signaling [25], [26]. Thus, actin polymerization is required for TCR-induced adhesion to these surfaces, emphasizing that signaling events downstream of the TCR are controlling this binding reaction.

**Figure 3:**
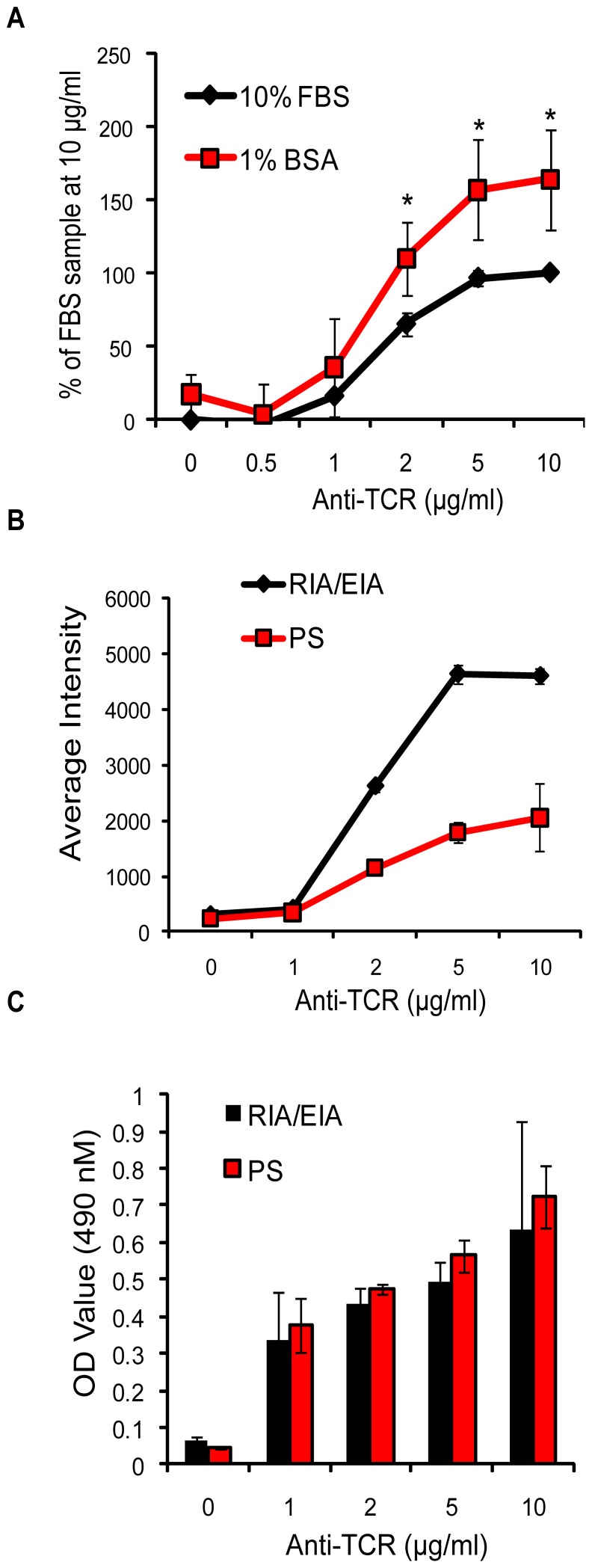
Non-covalent interactions facilitate adhesion to anti-TCR treated plates. (A) Labeled Jurkat cells were allowed to adhere to RIA/EIA plates coated with anti-TCR for 30 minutes in RPMI containing 1% BSA or 10% FBS. The data were normalized to the 10% FBS treatment at 10 µg/ml anti-TCR. The average of five experiments ± SD was then calculated and graphed. * p≤0.05. (B) RIA/EIA plates or polystyrene plates were coated with various doses of anti-TCR. Labeled Jurkat E6.1 cells were applied to these plates and incubated for 30 minutes. The average intensity of binding in triplicate wells ± SD was quantified and graphed. These data are representative of three experiments. (C) The amount of anti-TCR antibody bound to each plate from (B) was quantified by ELISA. The graph shows the average of triplicate wells ± SD and is representative of three independent experiments.

The data above demonstrate that TCR-induced adhesion required intracellular signaling that controls actin polymerization. However, it still was not clear how TCR stimulation was driving non-specific adhesion to these surfaces. These assays were performed in the presence of 10% fetal bovine serum (FBS), which may contain extracellular matrix (ECM) proteins that could activate integrin receptors expressed on T cells, leading to enhanced adhesion. Therefore, we repeated these experiments using RPMI media containing 1% bovine serum albumin (BSA). As shown in Figure 3A, adhesion at multiple doses of anti-TCR was significantly greater in the presence of BSA. These data suggest that adhesion to these anti-TCR-treated surfaces was not mediated by ECM proteins found in the FBS. Another potential explanation was that the chemistry of the RIA/EIA plate was mediating these effects, since these surfaces have significant hydrophobic and hydrophilic charge. Thus, TCR-induced adhesion could be enhanced by non-specific interactions between cell surface proteins and these plates. To investigate this possibility, Jurkat cells were stimulated on RIA/EIA plates or on polystyrene plates, which have hydrophobic but not hydrophilic surfaces. Interestingly, the cells bound better to RIA/EIA plates compared to polystyrene plates (Figure 3B), even though there were no overt differences in antibody binding as measured by ELISA (Figure 3C). Thus, TCR-mediated adhesion to these plates is driven by the magnitude of intracellular signaling that promotes actin polymerization and relies upon non-selective hydrophobic, hydrophilic, or charge-charge interactions between the plates and surface proteins on the T cells.

### Cell morphology and actin structure change following TCR activation

**Figure 4:**
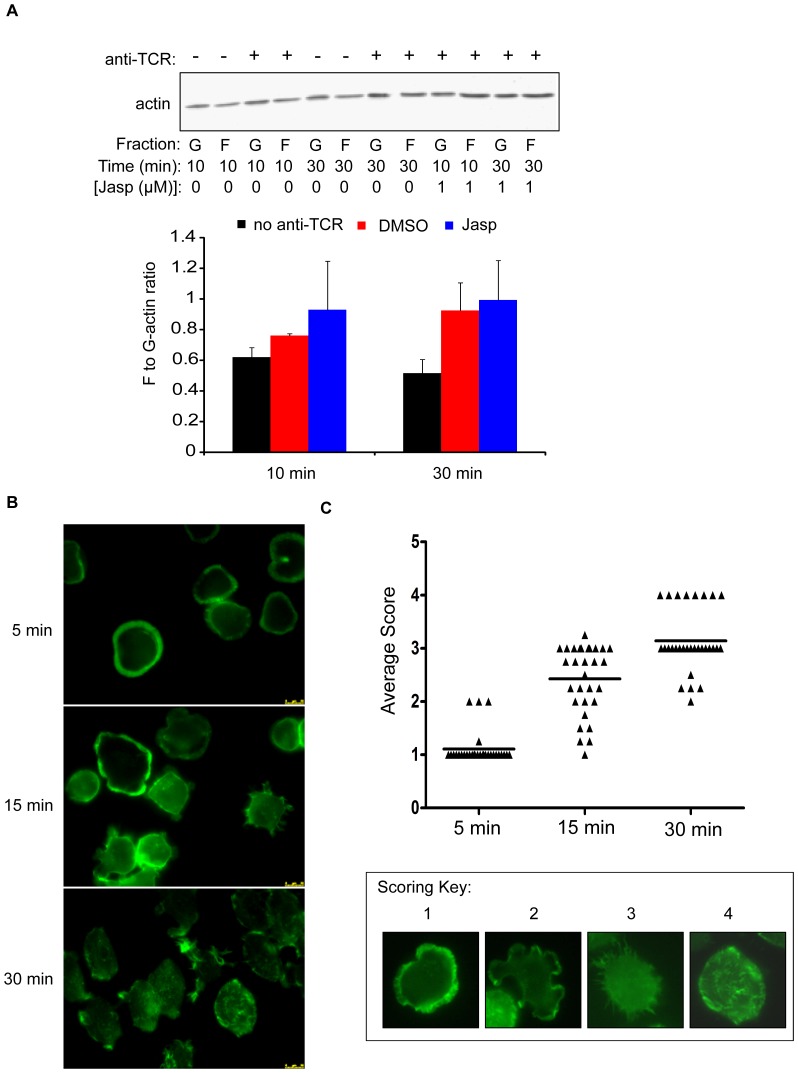
T cell morphology and actin structure differ between the early and late stages of TCR activation. (A) Jurkat cells were pre-treated with DMSO or jasplakinolide (Jasp) for 15 minutes at 37°C. These cells were then stimulated with or without plate-bound anti-TCR for the indicated times. Fractions containing G-actin and F-actin were isolated and then run on a polyacrylimide gel. Immunoblotting was used to detect changes in G-actin and F-actin. A representative blot is shown (top panel). The ratio of F-actin to G-actin was determined, and the average ratio from three independent experiments ± SEM was calculated and graphed (bottom panel). (B) Jurkat cells were stimulated with anti-TCR on plastic chamber slides for the indicated times. These cells were then stained using FITC-phallodin and imaged using epifluorescence. The phallodin images shown at each time point are representative of three experiments. Yellow scale bar = 10 µM. (C) The average blinded score of thirty individual cells at each time was determined and graphed. Examples of each scoring category are shown in the key below the graph.

The actin cytoskeleton regulates morphological changes that drive cellular adhesion [1], [2]. This suggests that differences in actin polymerization may explain why TCR-induced adhesion is enhanced over time. We therefore measured changes in G-actin and F-actin content by immunoblotting. Compared to the unstimulated cells, the F/G-actin ratio was only modestly increased in cells that were stimulated with anti-TCR for 10 minutes. By contrast, the F/G-actin ratio was approximately 2-fold higher than the unstimulated cells after 30 minutes of activation, and this change was similar to that induced by the actin polymerizing agent jasplakinolide (Figure 4A). Thus, actin polymerization increases over time following TCR induction.

To address if there were differences in actin structure over time, Jurkat cells were stimulated using immobilized anti-TCR coated onto plastic chamber slides. These cells were then stained using FITC-phallodin and imaged by epifluorescence. As shown in Figure 4B–4C, cells that were stimulated for 5 minutes were round in shape and formed lamellipodia that were rich in F-actin, consistent with previous reports that had utilized glass chamber systems [9], [10]. By contrast, cells that were activated for 30 minutes were more elongated and formed many actin structures, including lamellipodia, filopodia, and stress fibers (Figure 4B–4C). Some of these cells also contained punctate actin structures that resembled focal adhesions (Figure 4B–4C). Jurkat cells that were stimulated for 15 minutes showed an intermediate phenotype (Figure 4B–4C), which was interesting given that they also had intermediate levels of adhesion at this time (Figure 1A). Collectively, these data demonstrate that TCR activation differentially regulates actin polymerization, and thus cellular morphology, at early and late times to modulate the strength of T cell adhesion.

### Actin polymerization controls the late-phase of TCR-induced adhesion

**Figure 5:**
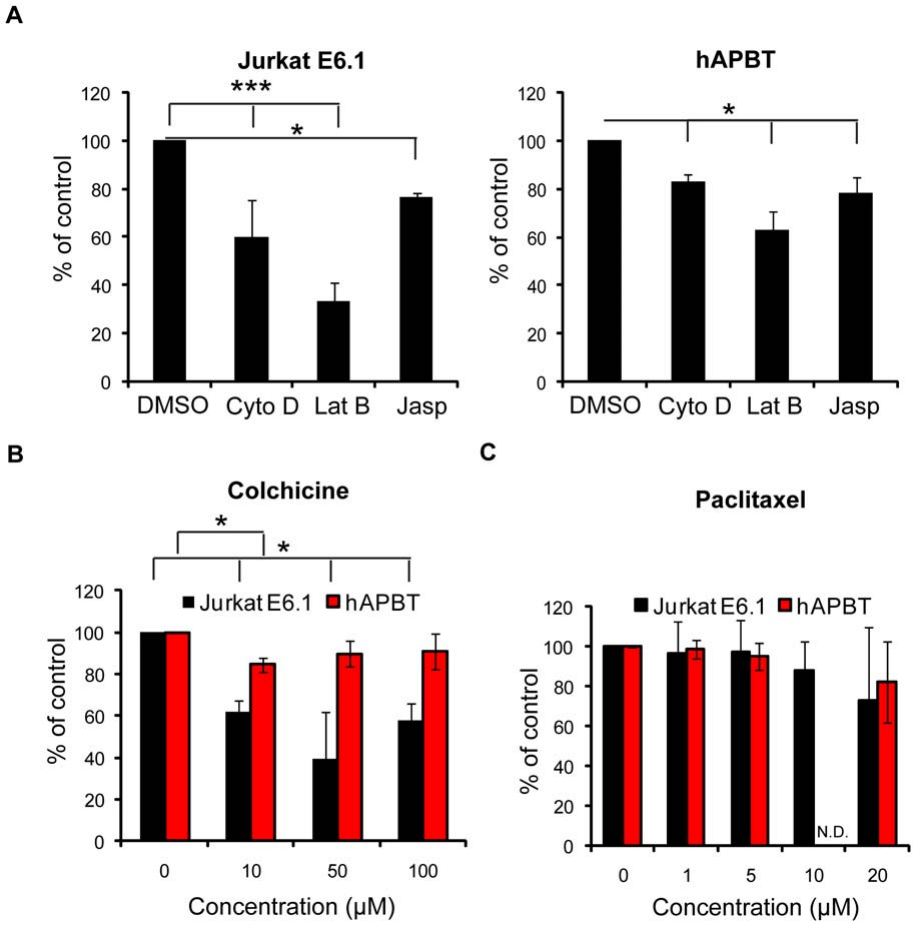
The actin cytoskeleton is necessary for late TCR-induced adhesion. (A) DMSO, Cytochalasin D (Cyto D), Latrunculin B (Lat B), or jasplakinolide (Jasp) was added to the stained Jurkat cells 15 minutes after beginning TCR stimulation on RIA/EIA plates for 30 minutes. The data were normalized to the DMSO controls, and the average of three independent experiments ± SD is depicted. * p≤0.05 *** p≤0.001(B) Jurkat E6.1 cells or hAPBTs were stained and treated with DMSO or the microtubule depolymerizing chemical colchicine for 30 minutes at 37°C. The DMSO samples were set to 100% and the average normalized data from three independent replicates ± SD was graphed. * p≤0.05 (C) Jurkat E6.1 cells or hAPBTs were treated and stimulated as in (B) but with the microtubule stabilizing agent paclitaxel. The normalized values from three independent replicates ± SD are shown graphically. N.D. demonstrates that no data were collected for this dose in hAPBTs.

Since several studies have examined the early signaling events that drive adhesion (reviewed in [1], [2]), we chose to focus on the intracellular signaling cascades that control the late stages of adhesion. We have recently shown that two waves of actin polymerization accompany soluble anti-TCR stimulation [18]. This second, smaller burst of actin remodeling peaks at 30–60 minutes after receptor activation. Interestingly, stimulation using immobilized anti-TCR antibodies also appeared to induce two unique stages of actin polymerization that correlated with two phases of human T cell adhesion (Figure 1A and Figure 4). Therefore, we first asked if the second phase of actin polymerization was required for the stabilized adhesion we observed at these later times. To address this question, cells were treated with cytochalasin D or latrunculin B 15 minutes after TCR stimulation had begun, immediately prior to the induction of the second burst of actin polymerization [18]. We found that Jurkat cell and hAPBT adhesion was significantly abrogated if these actin inhibitors were applied at this time (Figure 5A). The ability of cytochalasin D and latrunculin B to reduce TCR-mediated adhesion could reflect a requirement for dynamic actin reorganization or a need for actin filaments that act as a signaling scaffold. Therefore, cells were also treated with jasplakinolide, which also significantly reduced TCR-induced adhesion at this time (Figure 5A). This inhibition was substantially less pronounced than that achieved by pre-treating cells with these actin inhibitors prior to beginning the stimulation, however (Figure 2B). These results suggest that the early phase of actin polymerization is necessary to induce cellular adhesion, while the second round of actin remodeling is required to maintain stable cell contact following TCR activation.

Microtubules have also been implicated in controlling T cell adhesion to APC and were shown to regulate spreading on glass coverslips coated with anti-TCR [1], [2], [9]. However, a precise role for microtubules in TCR-mediated adhesion has not been investigated. We found that disrupting microtubules using colchicine or stabilizing microtubules with paclitaxel had little effect on the adhesion of hAPBTs. The binding of Jurkat cells was also unaffected by paclitaxel treatment but was more sensitive to colchicine treatment for an unknown reason (Figure 5B–5C). Collectively, these results demonstrate that actin, but not microtubule, reorganization is required for early and late TCR-induced adhesion.

### The activity of the Src family kinases is dispensable for late adhesive events downstream of the TCR

**Figure 6:**
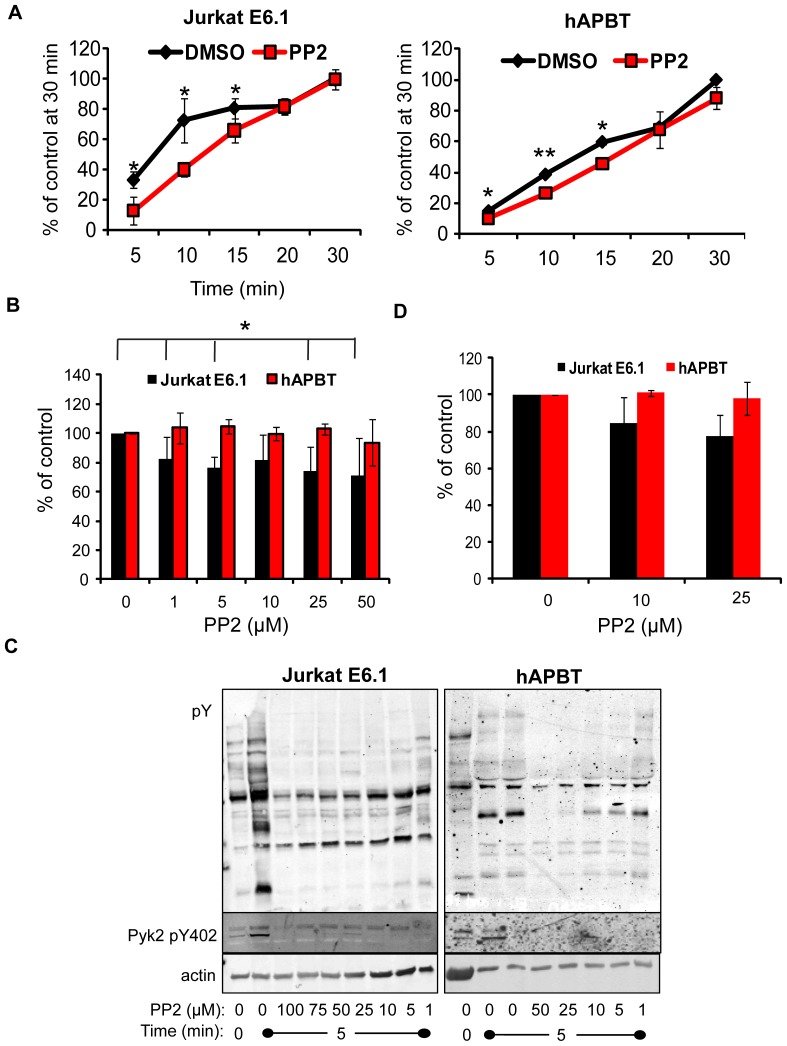
The enzymatic activity of the Src family kinases is needed for early but not late TCR-mediated adhesion. (A) Jurkat E6.1 cells (left panel) or hAPBTs (right panel) were stained and then incubated with 25 µM of the Src family kinase inhibitor PP2 for 15 minutes at 37°C. The cells were then stimulated on an anti-TCR coated RIA/EIA plate for various times. The data were normalized such that the 30 minute time point was set to 100%. The mean ± SD from three to four independent replicates for each stimulation time was then calculated and graphed. * p≤0.05 ** p≤0.01 (B) Jurkat E6.1 cells and hAPBTs were stained and then incubated with DMSO or the indicated doses of PP2 for 15 minutes. The cells were then stimulated with immobilized anti-TCR for 30 minutes. The amount of cellular binding was normalized to the DMSO sample. The Jurkat E6.1 graph shows the average of six independent experiments ± SD, and the hAPBT results are the mean of three independent experiments ± SD. * p≤0.05 (C) Jurkat E6.1 cells (left) and hAPBTs (right) were incubated with various doses of PP2 for 15 minutes and then stimulated with soluble anti-TCR antibody for five minutes or left unstimulated (US) as a control. Cellular lysates were resolved by PAGE and total changes in TCR-inducible tyrosine phosphorylation or Pyk2 tyrosine 402 phosphorylation were detected by immunoblotting. Actin was used as a loading control. (D) Jurkat cells or hAPBTs were stained and treated with PP2 for 15 minutes after beginning TCR stimulation using immobilized antibodies. After 30 minutes, the amount of adhesion was determined and normalized to the DMSO samples. The graph shows the average of three independent experiments ± SD.

TCR-dependent signaling is initiated by the SFK, Lck and Fyn [8], and the earliest phases of TCR-mediated adhesion require the activity of these kinases [1], [2]. To examine if SFK activity was required for adhesion, Jurkat cells and hAPBTs were pre-treated with the selective SFK inhibitor PP2 prior to stimulation on anti-TCR coated plates for various times. We verified that the early stages of adhesion that occurs between 5 and 15 minutes after TCR activation was dependent upon the enzymatic functions of Lck and/or Fyn. However, the late phase of adhesion did not require Lck and Fyn activity (Figure 6A). It is not likely that these differences are due to incomplete repression of SFK activity, as multiple doses of PP2 impaired TCR-mediated signaling (Figure 6C), while only having a modest effect on Jurkat cell binding and no effect on hAPBT adhesion (Figure 6B and 6D). To more definitively show that the second phase of adhesion was independent of SFK activity, Jurkat cells or hAPBTs were stimulated for 30 minutes with immobilized anti-TCR and treated with PP2 during the last 15 minutes of activation. In doing so, these cells only had SFK function inhibited immediately before the late wave of adhesion had begun. As shown in Figure 6D, inhibiting SFK activity at this time also had no effect on their ability to bind to these plates. Therefore, it appears that the early and late phases of TCR-induced adhesion are differentially controlled by the enzymatic activities of Lck and Fyn, suggesting that the first and second waves of adhesion are driven by distinct signaling pathways.

### Fyn is required for the late stage of TCR-induced adhesion

**Figure 7:**
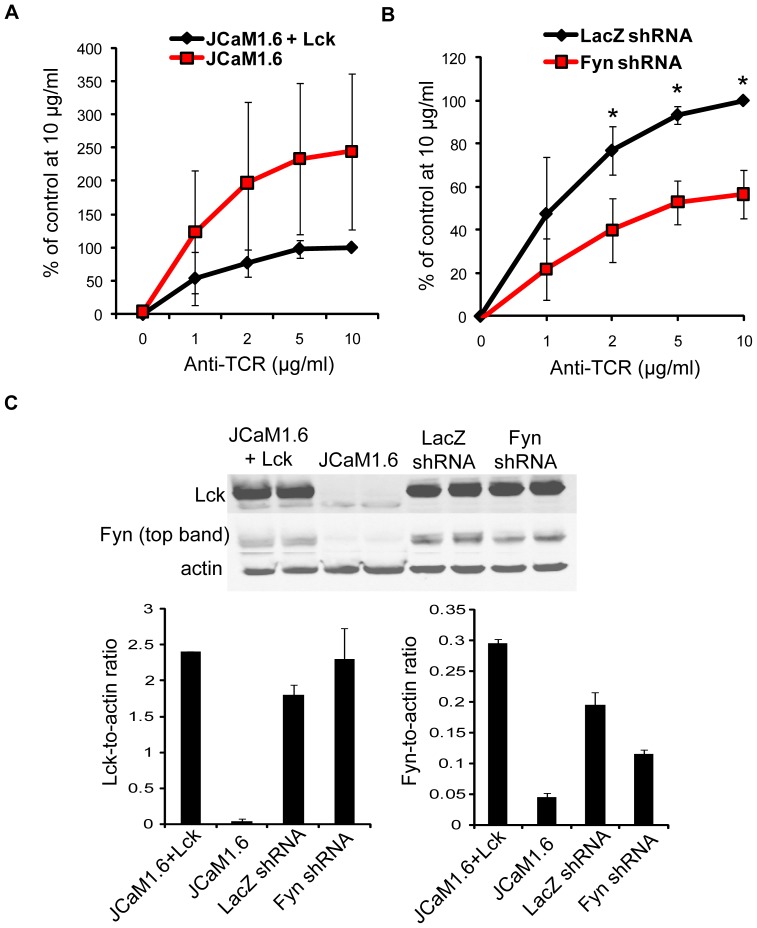
Fyn is necessary for TCR-mediated adhesion. (A) JCaM1.6 cells and JCaM1.6 cells that expressed Lck were stained and applied to an anti-TCR coated plate. The cells were allowed to adhere to the plate for 30 minutes at 37°C. The amount of binding was normalized as described in Materials and Methods, and the average of three independent experiments ± SD is shown. (B) Control (LacZ shRNA) and Fyn-deficient (Fyn shRNA) Jurkat cells were stained and stimulated with plate-bound anti-TCR for 30 minutes at 37°C. The data were normalized as described in Materials and Methods. These results are the mean of three independent experiments ± SD. * p≤0.05 (C) Whole cell lysates from JCaM1.6+Lck, JCaM1.6, LacZ shRNA-expressing Jurkats, and Fyn-deficient Jurkat cells were resolved by PAGE. Immunoblotting was used to detect Lck and Fyn. Actin was used as a loading control. The relative expression of each protein compared to actin also was quantified by densitometry. The average ratio of the duplicate bands ± SD is presented graphically.

Our results clearly show that the two phases of TCR-inducible adhesion differentially require SFK activity. Since Lck and Fyn both have SH2 and SH3 domains that make them capable of serving as protein scaffolds [27], we next asked if the deletion of Lck and Fyn impacted the late-stage of TCR-dependent adhesion. We first used JCaM1.6 cells, a Jurkat cell mutant that lacks Lck and expresses low levels of Fyn, or JCaM1.6 cells that were reconstituted with Lck (Figure 7C). Although not statistically significant, JCaM1.6 cells surprisingly bound to these plates better than the Lck-reconstituted cells (Figure 7A), potentially because Fyn was better able to control adhesion in the absence of Lck. To investigate if Fyn regulated adhesion, we next stimulated Jurkat cells that stably expressed a control shRNA against LacZ or a Fyn-specific shRNA (Figure 7C and [17]). The adhesion of Fyn-deficient Jurkat cells was decreased by approximately 40–50% when compared to the controls (Figure 7B). These data demonstrate that Fyn is required for the late adhesive events that occur following TCR activation. Furthermore, they suggest that the adaptor function of Fyn, but not its kinase activity, controls the second wave of adhesion.

### The PI3K/Akt and MEK pathways are not required for TCR-mediated adhesion

**Figure 8:**
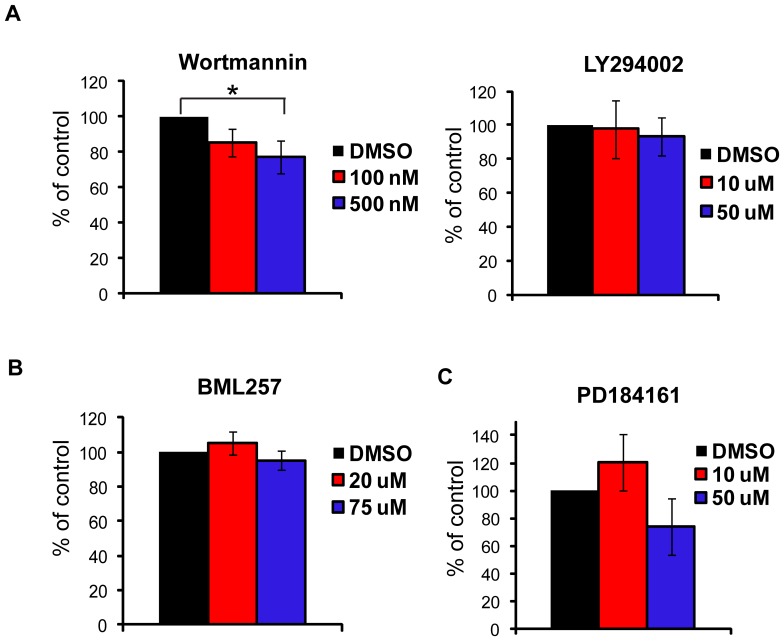
PI3K, Akt, and MEK signaling are not needed for late TCR-mediated adhesion. (A) hAPBTs were stained and pretreated with DMSO or the PI3K inhibitors wortmannin (left panel) or LY294002 (right panel) for 15 minutes at 37°C. The cells were then stimulated with plate bound anti-TCR for 30 minutes at 37°C. * p≤0.05 (B,C) hAPBTs were stained and pretreated with DMSO, the Akt inhibitor BML257 (B), or the MEK inhibitor PD184161 (C) for 30 minutes at 37°C. The cells were then stimulated with immobilized anti-TCR for 30 minutes at 37°C. All data were normalized such that DMSO was set to 100% and the results are the mean of three independent experiments ± SD.

Early TCR-induced actin polymerization is driven by PI3K, a protein that also controls adhesion mediated by some integrin receptors [1], [28]. To investigate if the enzymatic function of PI3K controls the late phase of TCR-mediated adhesion, we pre-treated hAPBTs with two PI3K inhibitors, wortmannin and LY294002, and stimulated them on plates coated with anti-TCR for 30 minutes. While the TCR-inducible phosphorylation of Akt and Erk1/Erk2 was inhibited by these treatments (data not shown and [17], [29]), the cellular binding was not significantly impacted by wortmannin or LY294002 pre-treatment, suggesting that PI3K activity was not required for the second wave of TCR-mediated adhesion (Figure 8A). Interestingly, the highest dose of wortmannin did modestly suppress binding, potentially because wortmannin inhibits myosin light chain kinase activity at this dose [30]. The inhibition of the serine/threonine kinase Akt, which is activated in a PI3K-dependent manner downstream of the TCR [29], also had no effect on hAPBT binding (Figure 8B). These results were not all that surprising, since SFK activity is required to phosphorylate LAT and SLP-76, proteins that control PI3K activation [8], [31]. We also found that inhibiting MEK signaling with PD184161 blocked Erk1/Erk2 phosphorylation but did not prevent hAPBT adhesion at the later time points (data not shown and Figure 8C). Thus, PI3K/Akt signaling and MEK kinase activation do not control the late-phase of TCR-induced adhesion.

### Pyk2 controls the late stage of TCR-mediated adhesion independently of its enzymatic activity

**Figure 9:**
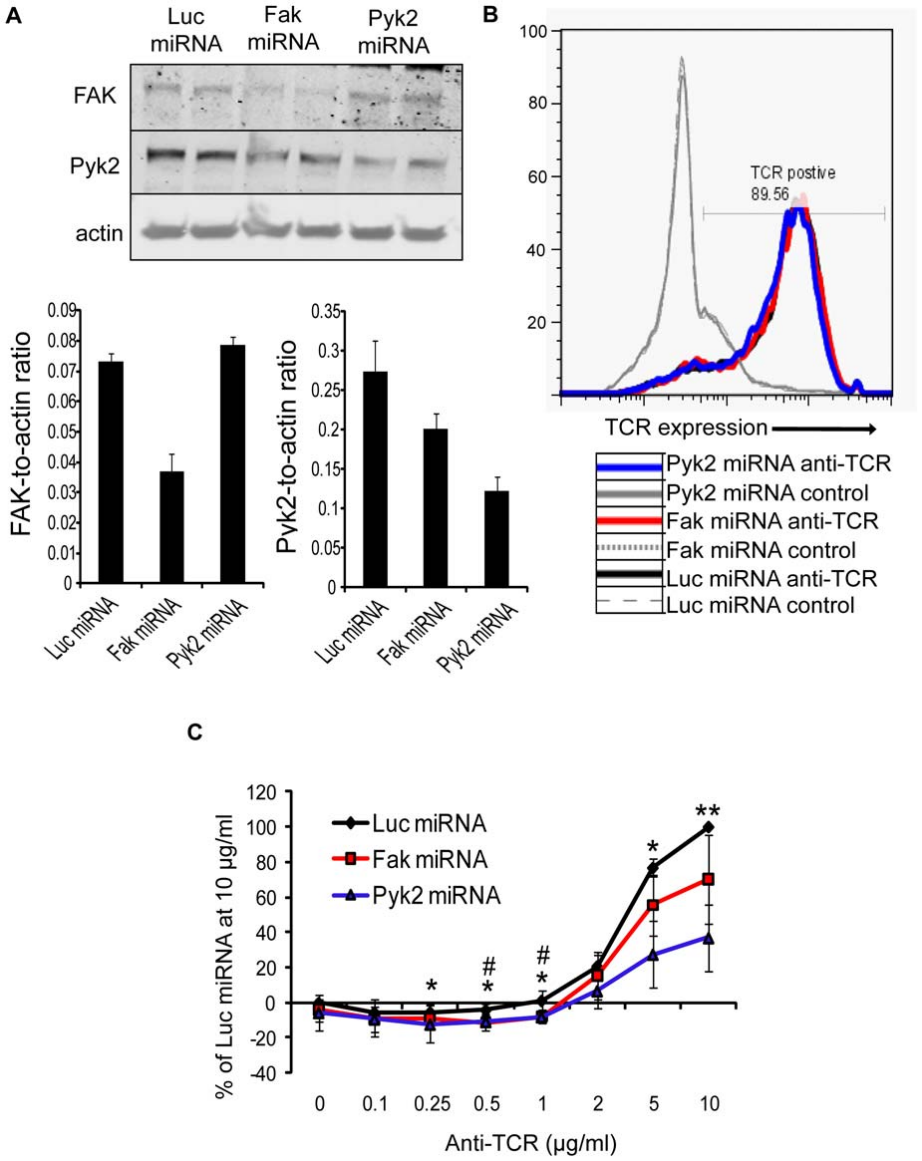
Pyk2 is required for late stage TCR-induced adhesion. (A) Jurkat E6.1 cells were transfected with the control luciferase (Luc), Fak, or Pyk2-specific microRNA (miRNA). 72 hours later, the cells were lysed and the expression of FAK, Pyk2, and actin was determined by immunoblotting. The graphs show the average ratio of FAK or Pyk2 expression relative to actin ± SD. (B) TCR expression was examined by flow cytometry. The histogram is representative of two independent experiments. (C) Transfected Jurkat cells were stained and stimulated with various doses of plate bound anti-TCR antibody for 30 minutes at 37°C. The data was normalized such that the Luc miRNA stimulation at 10 µg/ml was set to 100%. The results are the mean of four independent experiments ± SD. Statistical analyses were performed to compare the differences in adhesion between the Luc versus Pyk2 miRNA samples (* p≤0.05 ** p≤0.01) and the Luc versus Fak miRNA samples (# p≤0.05).

The related non-receptor tyrosine kinases FAK and Pyk2 are phosphorylated upon TCR activation [14], [17], [18] and have been linked to actin cytoskeletal reorganization downstream of many receptors [12], [18], [32]. Therefore, we next wanted to examine if FAK and Pyk2 were needed for the late-phase of TCR-induced adhesion. To that end, we transiently knocked down FAK and Pyk2 expression using microRNA-based targeting vectors, which suppressed FAK and Pyk2 protein expression by 50–60% (Figure 9A). TCR expression and total changes in TCR-dependent tyrosine phosphorylation are not impaired in the absence of FAK and Pyk2 (Figure 9B and data not shown). However, the adhesion of Pyk2-deficient Jurkat cells was significantly decreased after TCR activation when compared to the control cells treated with the Luciferase-specific (Luc) miRNA (Figure 9C). By contrast, cellular binding was not defective in the absence of FAK, although there was a statistically significant decrease in the adhesion of FAK-deficient cells at lower doses (0.5–1 µg/ml) of anti-TCR where binding was barely detectable (Figure 9C). These data suggest that Pyk2 but not its related kinase FAK is required for the late-stages of TCR-induced adhesion.

**Figure 10:**
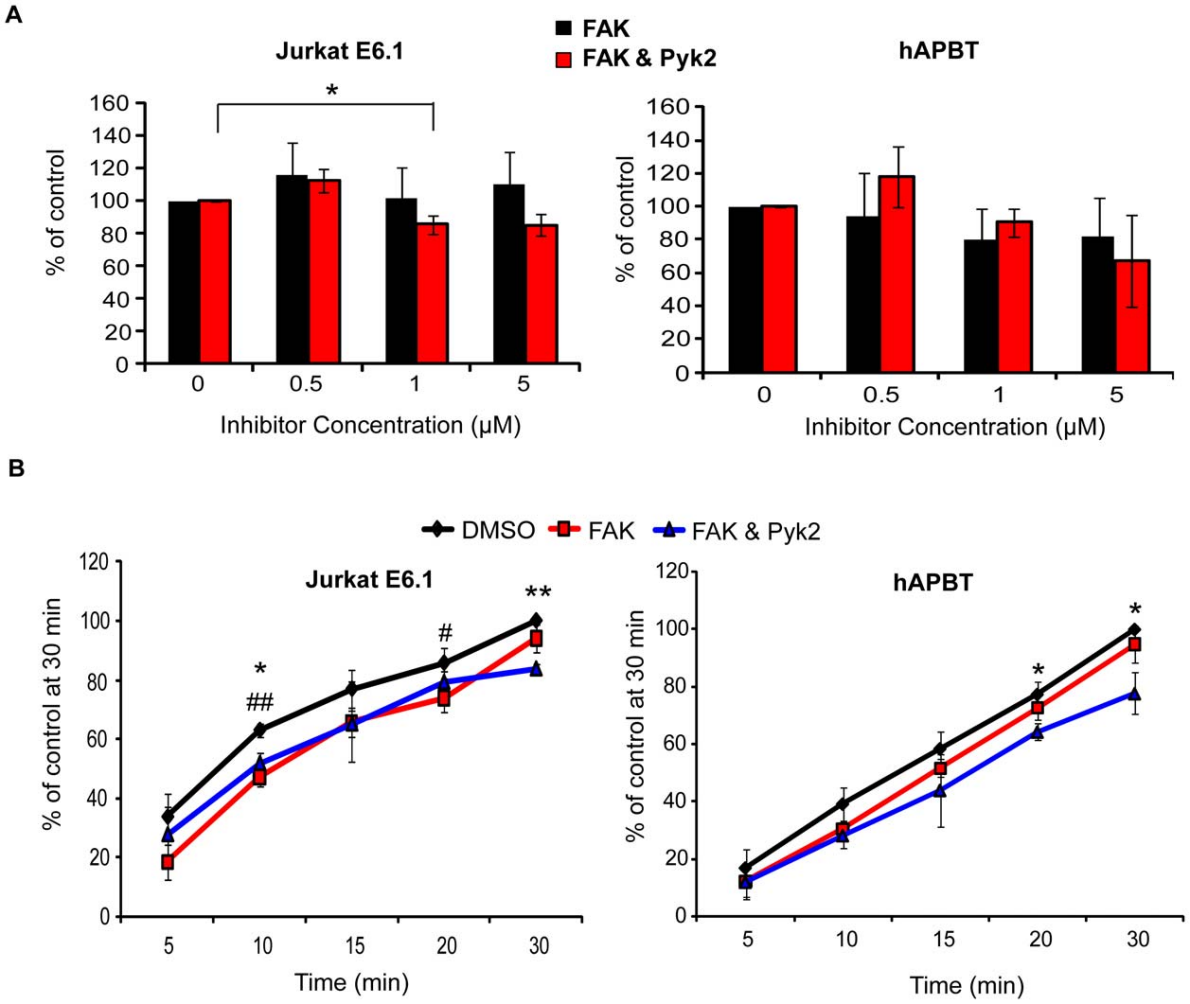
The enzymatic function of FAK and/or Pyk2 partially regulates TCR-mediated adhesion. (A) Jurkat E6.1 (left panel) or hAPBTs (right panel) were stained and pretreated with DMSO, a FAK inhibitor, or a FAK/Pyk2 dual inhibitor for 60 minutes at 37°C. The cells were then stimulated on an anti-TCR coated RIA/EIA plate for 30 minutes. The DMSO sample was set to 100% and the average normalized data from three independent experiments ± SD is shown. * p≤0.05 (B) Jurkat E6.1 cells (left panel) or hAPBTs (right panel) were stained and pre-treated with the 500 nM of the inhibitors as in (A) and stimulated with immobilized anti-TCR for various times. The DMSO sample at 30 minutes was set to 100% and the average normalized data from three independent experiments ± SD is shown. * p≤0.05 and ** p≤0.01 (DMSO sample vs. FAK/Pyk2 inhibitor sample); # p≤0.05 and ## p≤0.01 (DMSO sample vs. FAK inhibitor sample).

Following TCR induction, Pyk2 is phosphorylated on tyrosines 402 and 580, two sites that regulate the enzymatic activity of this kinase [12]. We have shown that Lck/Fyn activity is required for phosphorylating these sites downstream of the TCR in human T cells (Figure 6C and [17], [18]). However, the SFK inhibitor PP2 had little effect on the adhesion of Jurkat cells and hAPBTs (Figure 6), suggesting that the kinase activity of Pyk2 is not required for TCR-mediated adhesion at these later times. To confirm these results, we pre-treated Jurkat cells or hAPBTs with a FAK kinase inhibitor or an inhibitor that blocks the enzymatic activities of both FAK and Pyk2 [32], [33]. Treatment with these inhibitors was sufficient to reduce TCR-induced FAK tyrosine 397 autophosphorylation but had no dramatic effect on overall tyrosine phosphorylation (data not shown). We found that inhibiting the kinase function of FAK or FAK and Pyk2 in combination had no major effect on the ability of either Jurkat cells or hAPBTs to bind after 30 minutes of TCR activation (Figure 10A–10B). Since Lck and/or Fyn activity was required for early TCR-induced adhesion (Figure 6A), we also examined whether the enzymatic function of FAK or Pyk2 was needed for adhesion at earlier times. As shown in Figure 10B, early TCR-induced adhesion was modestly reduced when both Jurkat cells and hAPBTs cells were treated with the FAK or FAK/Pyk2 inhibitor. This suggests that the enzymatic activity of FAK and/or Pyk2 partially controls TCR-mediated signals that drive adhesion within the first 20 minutes following receptor activation. Collectively, these results demonstrate that Pyk2 regulates TCR-induced adhesion in a manner that is largely independent of its kinase activity.

## Discussion

**Figure 11:**
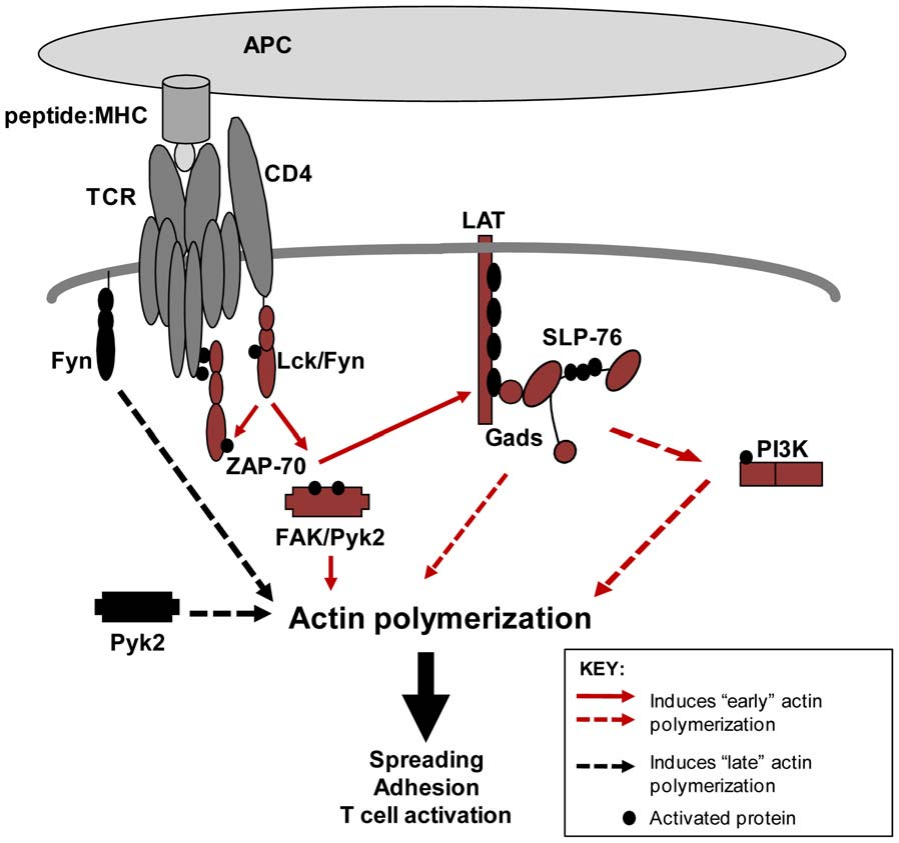
Proposed model for TCR-mediated adhesion in human T cells.

In this manuscript, we have identified two separate waves of TCR-induced adhesion using a near-infrared-based adhesion assay. We found that both the first and second phases of adhesion required actin polymerization. However, these two stages appear to be driven by distinct intracellular signaling pathways, since the first and second waves differentially required Lck/Fyn activity. We also reveal novel, non-enzymatic functions for Fyn and Pyk2 in controlling this second period of adhesion. A summary of these results is shown in Figure 11. Together, these data demonstrate that the canonical TCR-induced signaling cascade does not solely regulate adhesion and emphasize that differences in kinetics profoundly influence the results obtained from T cell adhesion studies.

The development and use of the modified plate-bound adhesion assay was critical for obtaining the results in this study. Plate-bound adhesion assays have long been utilized to examine how TCR activation drives T cell adhesion to integrin ligands like ICAM-1, VCAM-1, or fibronectin. However, these experiments are problematic for examining which TCR signaling pathways control adhesion, as these ligands also induce intracellular signaling that leads to adhesion [22], [23]. They also employ soluble TCR-specific antibodies, thereby reducing the need for dynamic, polarized cytoskeletal changes to induce TCR signaling. In contrast, we used immobilized anti-TCR antibodies, which better mimic the T cell-APC or T cell-target cell interaction that subsequently promotes TCR activation. We showed that adhesion in this system was not an artifact of high affinity antibody binding to the TCR or F_c_γ receptors (Figure 2). Interestingly, the TCR-stimulated cells bound best to highly hydrophobic and hydrophilic surfaces (Figure 3B). These data suggest that adhesion was driven by interactions that non-specifically emulate the binding of adhesion receptors with their ligands. Thus, signaling downstream of single receptors like the TCR can be studied in this system while maintaining the chemistry of protein-protein interactions that occur during cell-cell contact.

Our results demonstrate that TCR-dependent adhesion is mediated by both an early SFK-dependent and a late SFK-independent signaling pathway (Figure 6). The early requirement for SFK activity is consistent with previous publications showing that signaling downstream of the LAT complex is critical for actin polymerization that drives T cell adhesion (reviewed in [1], [2]). However, TCR-induced actin remodeling and adhesion has been reported to occur in cells that lack functional LAT or SLP-76 [9], [34], findings that support the idea that adhesion can occur independently of SFK activity. Although it is not precisely known how adhesion is occurring without the catalytic functions of Lck and/or Fyn, it is possible that the protein Nck plays a role in this process. Upon TCR stimulation, Nck associates with phosphorylated SLP-76, which in turn promotes actin polymerization via activation of WASp and Arp2/3 [1], [2], [5]. While still controversial, Nck has also been reported to directly associate with the TCR in the absence of ITAM phosphorylation, suggesting that SFK activity was not needed for this process [35]–[37]. Recent studies have demonstrated that Nck could bind to the guanine nucleotide exchange factor Vav1 independently of SLP-76 and that this interaction was needed for actin polymerization [38], [39]. Therefore, it would be interesting to see if the Nck-Vav1 association at the TCR complex was required for the late, SFK-independent stage of adhesion we observed in these studies.

We also demonstrated that the non-catalytic functions of the tyrosine kinases Fyn and Pyk2 were required for the late period of TCR-induced adhesion (Figures 7, 9, and 10). These results were unexpected, since Pyk2 phosphorylation by Lck and/or Fyn and actin polymerization downstream of the TCR are coincident [18]. How, then, might Fyn and Pyk2 control actin polymerization at these late times independently of their kinase activity? It is feasible that these kinases serve as scaffolds to recruit actin-binding and cytoskeletal-remodeling proteins to sites of adhesion at these later times. Pyk2 has been demonstrated to constitutively associate with the actin-binding protein paxillin at the MTOC in murine CD8 T cells [20], and the MTOC and Pyk2 are simultaneously recruited to the T cell-APC contact site, a location that also contains T cell adhesion molecules [21], [40], [41]. Interestingly, a kinase dead mutant of Pyk2 could still localize to this site [21], consistent with our results showing that the non-enzymatic function of Pyk2 controls adhesion (Figures 9 and 10). The observation that TCR stimulation drives the formation of filopodia, stress fibers, and/or lamellipodia over time suggests that Rho family GTPase activation may vary at different times after TCR activation (Figure 4). Interestingly, Pyk2 has been demonstrated to interact with Vav1 when overexpressed in Jurkat cells [42], and the Vav1-Pyk2 interaction was shown to regulate Rho activation downstream of β_3_ integrins [43]. Fyn was found to control Rac activation in mast cells [44], a result that is interesting given that TCR-induced Rac activation can occur independently of SLP-76 under certain conditions [34]. Therefore, Fyn and Pyk2 may control Rho family GTPase activation at adhesive sites, thereby driving localized actin polymerization to stabilize binding in response to ongoing TCR stimulation.

Collectively, the findings presented here demonstrate that TCR signaling induces two distinct phases of adhesion in human T cells. We predict that these two pathways are essential for modulating the strength of adhesion between T cells and antigen-bearing cells. First, the canonical TCR signaling pathway would promote dramatic reorientation of the cytoskeleton, which rapidly induces weak adhesion between APC or infected target cells and T cells. The late, non-enzymatic pathway controlled by Fyn and Pyk2 would then continue to induce less pronounced, localized actin rearrangements that help stabilize contacts with these cells. Importantly, the proximal signaling machinery would then be available to continuously activated TCRs and co-stimulatory molecules such that gene transcription could be induced. The use of this non-enzymatic pathway may also position Fyn and Pyk2 at sites of adhesion, allowing these kinases to participate in signaling that further boosts actin polymerization and binding downstream of adhesion receptors. Thus, the coordinated actions of these distinct adhesive pathways would serve to promote stabilized cell-cell contacts, while simultaneously inducing T cell effector responses that regulate both infectious and pathogenic human diseases.

## Materials and Methods

### Ethics Statement

All experiments using primary human T cells were conducted in accordance with the Declaration of Helsinki. Peripheral blood mononuclear cells (PBMCs) were acquired from completely anonymous donors that had consented for blood donation at the DeGowin Blood Center at the University of Iowa. These donors also provided written consent for their blood cells to be used by investigators at the University of Iowa in research projects that required human blood products. This consent form has been reviewed and approved by the Institutional Review Board (IRB) at the University of Iowa. To protect the privacy of the donors who had provided written consent, the PBMCs that were provided to the investigators in this study were completely de-identified. Therefore, the specific studies described in this manuscript were exempt from the University of Iowa's IRB guidelines on the ethical use of human samples.

### Reagents

RPMI 1640, L-glutamine, penicillin-streptomycin, and 1× phosphate buffered saline (PBS) were obtained from Gibco. The anti-CD3 antibody (clone OKT3), the unconjugated and PE-conjugated anti-αβ TCR antibodies (clone IP26), the anti-CD28 (clone CD28.2), and anti-CD4 antibody (clone RPA-T4) were purchased from Biolegend. The anti-phospho-tyrosine antibody (clone 4G10), anti-actin antibody (clone C4), anti-FAK antibody, and PVDF-FL membranes were obtained from Millipore. The magnetic Dynabeads and the anti-phospho Pyk2 tyrosine 402 antibody were bought from Invitrogen. The anti-Pyk2 antibody (clone YE353) was from Abcam. The CellVue Burgundy cell labeling kit, near-infrared conjugated secondary antibodies, and anti-β-actin antibody were purchased from Licor Biosciences. The HRP-conjugated anti-mouse secondary antibody was acquired from Jackson Immunoresearch, and the TMB Peroxidase Substrate Solutions were obtained from KPL. The Nucleofector Solution Kit V was purchased from Lonza. The 96 well EIA/RIA flat-bottom plates were from Costar. Cytochalasin D, jasplakinolide, and PP2 were obtained from Calbiochem. The FAK inhibitor (PF573228) and the FAK/Pyk2 inhibitor (PF431396) were purchased from Tocris Bioscience. Cayman Chemical Company was the source of the wortmannin, BML257, LY294002, PD184161, and colchicine. Paclitaxel and FITC-phallodin were acquired from Sigma, and latrunculin B was obtained from Biomol International. The recombinant human IL-2 was obtained from the Research and Reference Reagent Program, Division of AIDS, NIAID, NIH. All chemicals used in these studies were research grade and were obtained from various sources.

### Generation of hAPBTs

Whole blood was obtained from anonymous donors between the ages of 18 and 55 years and passed through leukocyte reducing system (LRS) cones [45]. PBMCs were obtained from these cones by washing them with isolation buffer (PBS containing 2 mM EDTA and 2% FBS) followed by Ficoll density centrifugation. The PBMCs were then incubated in complete RPMI media (RPMI 1640 supplemented with 10% FBS, 2 mM L-glutamine, and 50 µg/ml streptomycin-50 U/ml penicillin) for 2–4 hours to remove adherent cells. The non-adherent cells were then cultured at 2–4×10^6^ cells/ml in complete RPMI supplemented with 100 U/ml recombinant human IL-2. To enrich for T cells, these cells were activated with magnetic Dynabeads coated with anti-CD3 and anti-CD28 for 5–7 days before use.

### Suppression plasmids

The pENTR miR30 expression vector containing the Luc miRNA was a gift from Dr. Anton McCaffrey and has been described previously [46]. We replaced the Luc targeting sequences with those against human *Pyk2* (forward 5′ cgtgcgtcatgaagttcttcaa; reverse 5′ ttgaagaacttcatgacgcact) or human *Fak* (forward 5′ cgatggcacagaagctattgaa; reverse 5′ ttcaatagcttctgtgccatct). These sequences, including the human U6 promoter, were also amplified by PCR using primers containing *Cla*I and *Xma*I restrictions sites or with a primer containing the *MFe*I recognition site. They were then ligated into the pVetLeGFP vector developed by the University of Iowa's Gene Vector Transfer Core. The final modified pVetLeGFP expression vectors that were used in transfection experiments contained two copies of the miR30 suppression sequences against Luc, Pyk2, and FAK that were independently driven by the human U6 promoter.

### Cell Culture and Transfections

Jurkat E6.1 cells, JCaM1.6 cells, JCaM1.6 cells reconstituted with Lck, and Jurkat cells expressing the LacZ or Fyn-specific shRNA have been previously described [17], [47], [48]. These cells were grown in complete RPMI media and maintained at a density of 3–5×10^5^ cells/ml. Where indicated, 5×10^6^ cells Jurkat E6.1 cells were transfected with 5 µg of microRNA plasmid using the Nucleofector Solution Kit V (Lonza) and the Amaxa Program X-001. The cells were cultured in complete RPMI for three days prior to use.

### Plate-bound adhesion assay

RIA/EIA plates were treated with various concentrations of anti-TCR diluted in PBS and incubated overnight at 4°C. The plates were then washed three times with PBS and blocked using 1% BSA prepared in PBS for 2 hours at 37°C. The Jurkat E6.1 cells or hAPBTs were stained following the protocol provided with the Licor CellVue Burgundy kit. Briefly, Jurkat E6.1 cells or hAPBTs were resuspended at 2×10^7^ cells/ml in Diluent C and stained with 4×10^−6^ M dye stock for 3.5 minutes or 3 minutes, respectively. These cells were then washed three times with excess complete RPMI to remove unbound dye. The blocked plates were washed three times using PBS, and 1.5–2×10^5^ cells in 100 µl of complete RPMI were applied to each well and incubated for 30 minutes at 37°C. For the time course experiments, 50 µl of complete RPMI was placed in each well following blocking. A 50 µl sample of the stained cells in complete RPMI was then added to each well and incubated for the indicated times. The plates were washed twice using PBS, resuspended in 100 µl of PBS, and viewed using the Licor Odyssey Infrared Imager.

### Inhibitor Experiments

RIA/EIA plates were coated with 5 µg/ml of anti-TCR (OKT3) and blocked as described above. Jurkat E6.1 cells or hAPBTs were stained as above and resuspended at 2×10^6^ cells/ml in complete RPMI. The cells were then treated with cytochalasin D (5 µM), latrunculin B (10 µM), and the indicated doses of BML257 or PD184161 for 30 minutes at 37°C. PF573228 and PF431396 were applied to the cells for 60 minutes at 37°C prior to stimulation. Where indicated, the cells were treated with the indicated doses of wortmannin, LY294002, and PP2 or 1 µM jasplakinolide for 15 minutes at 37°C before stimulation on the anti-TCR treated plates. These cells were then added to the plates and incubated for 30 minutes as described above. For the post-treatment experiments, Jurkat E6.1 cells and hAPBTs were treated for 15 minutes with DMSO, cytochalasin D (5 µM), latrunculin B (10 µM), jasplakinolide (1 µM), or PP2 (10 µM or 25 µM) after beginning stimulation using immobilized anti-TCR.

### Flow Cytometry

1×10^6^ cells were washed in FACS buffer (PBS+5% FBS+0.5% sodium azide) and resuspended at 1×10^7^ cells/ml in FACS buffer. The cells were then stained on ice for 30 minutes using the PE-conjugated αβ TCR antibody. Following extensive washing with FACS buffer, equal numbers of live cell events were collected using the Accuri flow cytometer. The mean fluorescence intensity (MFI) of the TCR for each sample was determined using the Cflow Plus Software. Alternatively, samples were collected using a Guava flow cytometer (Millipore) and analyzed using FlowJo.

### Data Analysis and Statistics

The intensity of the membrane dye was quantified using the Odyssey v3 software and exported into Microsoft Excel. For each experiment, the average triplicate intensity for each treatment was determined using Microsoft Excel and reported as the average triplicate intensity ± standard deviation where indicated. Alternatively, the mean triplicate intensity from individual experiments was normalized, and the average normalized intensity ± standard deviation was calculated. The formulas used to normalize the data are shown below.

The equation used to calculate the normalized intensity for the inhibitor experiments:

(1) % of DMSO control = [(Average Intensity of inhibitor treatment−Average Intensity of DMSO sample at 0 µg/ml)÷(Average Intensity of DMSO sample−Average Intensity of DMSO at 0 µg/ml)]×100%.

The formula used to calculate the normalized intensity of the microRNA experiments:

(2) % of Luc control = [(Average Intensity treatment−Average Intensity of Luc miRNA at 0 µg/ml)÷(Average Intensity of Luc miRNA at 10 µg/ml−Average Intensity of Luc miRNA at 0 µg/ml)]×100%.

To account for differences in TCR expression between the Jurkat variants with or without Lck or Fyn, the average triplicate intensity from each experiment was normalized to the surface TCR expression with the following formula:

(3) Normalized intensity = average triplicate intensity÷MFI of TCR

The results from (3) was then used to calculate the relative change in adhesion compared to the control:

(4) % of control = [(Normalized Intensity of treatment−Normalized Intensity of control cells at 0 µg/ml)÷(Normalized Intensity of control cells at 10 µg/ml−Normalized Intensity of control cells at 0 µg/ml)]×100%.

All statistics were performed in Microsoft Excel using a two-tailed *t*-test assuming unequal variance, and p-values that were calculated relative to the controls are denoted in the figure legends.

### Cell Stimulation and Immunoblotting

Jurkat E6.1 cells or hAPBTs were stained and pre-treated with the inhibitors as described above. For each stimulation, 1×10^6^ Jurkat cells or hAPBTs were centrifuged and washed with RPMI 1640 without supplements. The cells were then resuspended at 2×10^7^ cells/ml in RPMI 1640 without supplements. The Jurkat E6.1 cells were stimulated using 2 µg/ml of soluble OKT3 for 5 minutes, while the hAPBTs were placed on ice and incubated with 2 µg/ml OKT3 and 2 µg/ml anti-CD4 for 30 minutes. The hAPBTs were then heated to 37°C for 10 minutes and stimulated for 5 minutes with 25 µg/ml anti-mouse IgG antibody. All samples were lysed using 50 µl of hot 2× lysis buffer (20 mM Tris pH 8.0, 2 mM EDTA, 2 mM Na_3_VO_4_, 20 mM DDT, 2% SDS, and 20% glycerol) and heated to 95°C for 4 minutes. The samples were then sonicated and loaded onto Criterion 4–15% polyacrylimide gels (Biorad). The proteins were transferred to PVDF-FL membranes. These membranes were blocked in 1% BSA-PBS for 1 hour at room temperature and incubated with anti-phospho-tyrosine or anti-phospho-Pyk2 tyrosine 402 for 2 hours at room temperature. Secondary anti-rabbit and anti-mouse antibodies were added for 30 minutes at room temperature, and the immunoblots were developed using the Licor Odyssey Infrared Imager. All blots were also probed using anti-actin to ensure equivalent protein loading.

### Antibody Binding ELISA

RIA/EIA or polystyrene plates were coated with various doses of OKT3 diluted in PBS overnight at 4°C. The plates were then rinsed using wash buffer (PBS supplemented with 0.05% Tween 20) and blocked for 2 hours in 1% BSA-PBS at 37°C. After extensive washing, HRP-conjugated anti-mouse IgG was added to the plates and incubated for 1 hour at 37°C. The plates were next washed and incubated with TMB peroxidase detection solution for 5 minutes, and the reaction was stopped using 0.67 M sulfuric acid. The OD values at 490 nM were then measured using a spectrophotometric plate reader (Biotek).

### Cellular Imaging

Permanox chamber slides (Lab-Tek) were coated with 5 µg/ml of anti-CD3 (OKT3) overnight at 4°C in PBS. The chambers were then washed extensively with PBS and equilibrated using 1 ml of chamber buffer (RPMI 1640 without phenol red, 10% FBS, 10% FBS, 2 mM L-glutamine, and 50 µg/ml streptomycin-50 U/ml penicillin) for 30 minutes at 37°C. The buffer was then removed and replaced with 300 µl of chamber buffer. Jurkat cells were washed once with PBS, and 2.5×10^5^ cells in 100 µl of chamber buffer were added to each well. The cells were stimulated at 37°C for the indicated times. The cells were then fixed using 4% methanol-free formaldehyde (Thermo Scientific) prepared in PBS for 30 minutes at room temperature. After washing twice with PBS, the cells were permeablized for 5 minutes using 0.25% Triton X-100 prepared in PBS. The cells were washed with PBS, placed into SEA BLOCK buffer (diluted 1∶3 in PBS), and stained using 200 nM FITC-phallodin for 2 hours at 37°C. The images were collected using the GFP-epifluorescence channel on the Lonza TIRF microscope found the Central Microscopy Core Facility at the University of Iowa. All images were taken using the 100× oil objective.

### Microscopy Analysis

For each time point, thirty randomly-selected cells from three independent experiments (∼10 per experiment) were independently scored by four blind reviewers. The scoring system was as follows: (1) Round cells with a contiguous, circular ring of actin along the outer edge; (2) irregular-shaped cells with broad, actin-rich protrusions; (3) circular cells with actin puncta and narrow, cilia-like protrusions; (4) oval-shaped cells that contain stress fibers and actin puncta but have few extensions. Representative images of these categories may be found in Figure 4. The average score for each cell was then calculated and plotted using GraphPad Prism 5.

### F/G-actin ratios by immunoblotting

24-well tissue culture-treated plates (Corning) were coated with 5 µg/ml anti-CD3 (OKT3) in PBS overnight at 4°C and then washed three times with PBS to remove unbound antibody. 1×10^6^ cells were placed into 100 µl of complete RPMI media and pre-treated for 15 minutes with DMSO or 1 µM jasplakinolide at 37°C prior to stimulation with the immobilized anti-CD3. After stimulation, the plates were centrifuged at 1200 rpm for 5 minutes, and the complete RPMI media was removed. 100 µl of actin stabilization buffer (0.1 M PIPES pH 6.9, 30% glycerol, 5% DMSO, 1 mM MgSO_4_, 1 mM EGTA, 1% Triton X-100, 1 mM ATP, and 1 EDTA-free protease inhibitor tablet (Roche)) was added to the wells. The plate was then incubated on ice for 10 minutes. After removing the adherent cells by scraping, the samples were centrifuged for 75 minutes at 13000 rpm. The supernatants containing G-actin were transferred to new tubes, and the pellet containing F-actin was solubilized using actin depolymerization buffer (0.1 M PIPES pH 6.9, 1 mM MgSO_4_, 10 mM CaCl_2_, and 5 µM cytochalasin D) for 1 hour on ice. 100 µl of hot 2× lysis buffer was then added to the fractions containing G-actin and F-actin. These samples were boiled for 4 minutes at 95°C and sonicated to reduce viscosity. 20 µl of these samples were loaded onto a gel and immunoblotting was conducted with an anti-β-actin antibody. The densitometric value for the G-actin and F-actin fractions was measured and expressed as the ratio of F-actin to G-actin for each sample. The average ratio ± SEM from three independent experiments was then calculated and graphed.
